# Correction to: Knockdown of FOXO3a induces epithelial-mesenchymal transition and promotes metastasis of pancreatic ductal adenocarcinoma by activation of the β-catenin/TCF4 pathway through SPRY2

**DOI:** 10.1186/s13046-021-02033-2

**Published:** 2021-08-09

**Authors:** Jun Li, Rumeng Yang, Yuting Dong, Manyao Chen, Yu Wang, Guoping Wang

**Affiliations:** 1 Institute of Pathology, Tongji Hospital, Tongji Medical College, Huazhong University of Science and Technology, 1095 Jiefang Dadao, Wuhan, 430030 People’s Republic of China; 2 Department of Pathology, School of Basic Medicine, Tongji Medical College, Huazhong University of Science and Technology, Wuhan, 430030 People’s Republic of China

**Correction to: J Exp Clin Cancer Res 38, 38 (2019)**

**https://doi.org/10.1186/s13046-019-1046-x**

Following publication of the original article [1], minor errors were identified in the images presented in Figs. 2, 4, 6 and 8; specifically:

- Fig. 2b: migration transwell assay of siCtrl SW1990 cells group
- Fig. 4a: wound healing assay of Vector PANC-1 at 0 h
- Fig. 4a: wound healing assay of siSPRY2 SW1990 at 0 h
- Fig. 6b: invasion transwell assay of siCtrl PANC-1 cells group
- Fig. 6c: Western blot of β-actin in both PANC-1 and SW1990 cells group
- Fig. 8: invasion transwell assay of Vector SW1990 cells group

**Fig. 2:**
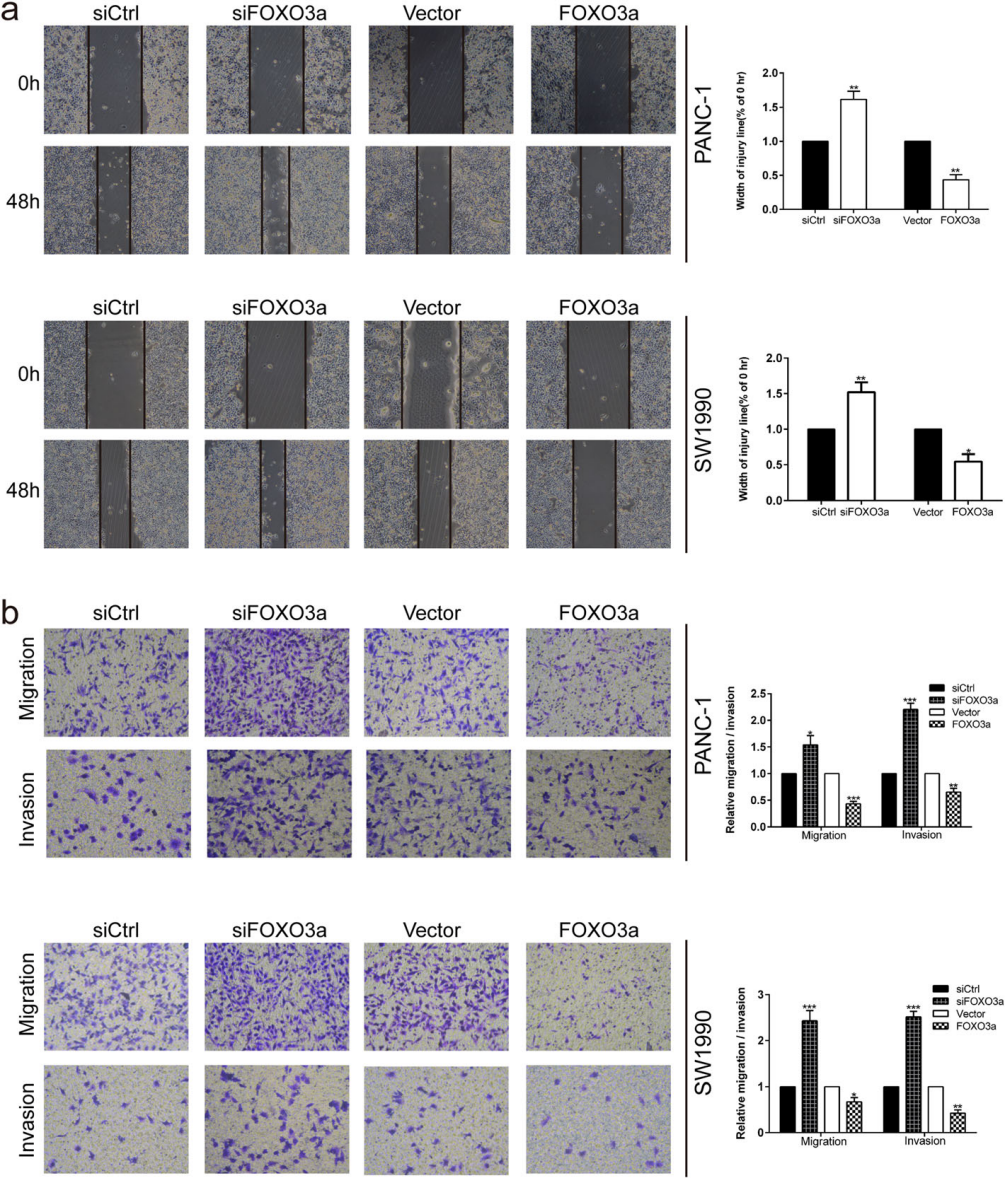
FOXO3a knockdown promoted the migration and invasion of PDAC cells. **a** Wound healing assay was carried out to investigate the migratory ability of PANC-1 and SW1990 cells. **b** Transwell migration and invasion assays were applied to assess the migratory and invasive capacities of PANC-1 and SW1990 cells. *P < 0.05, **P < 0.01, ***P < 0.001

**Fig. 4:**
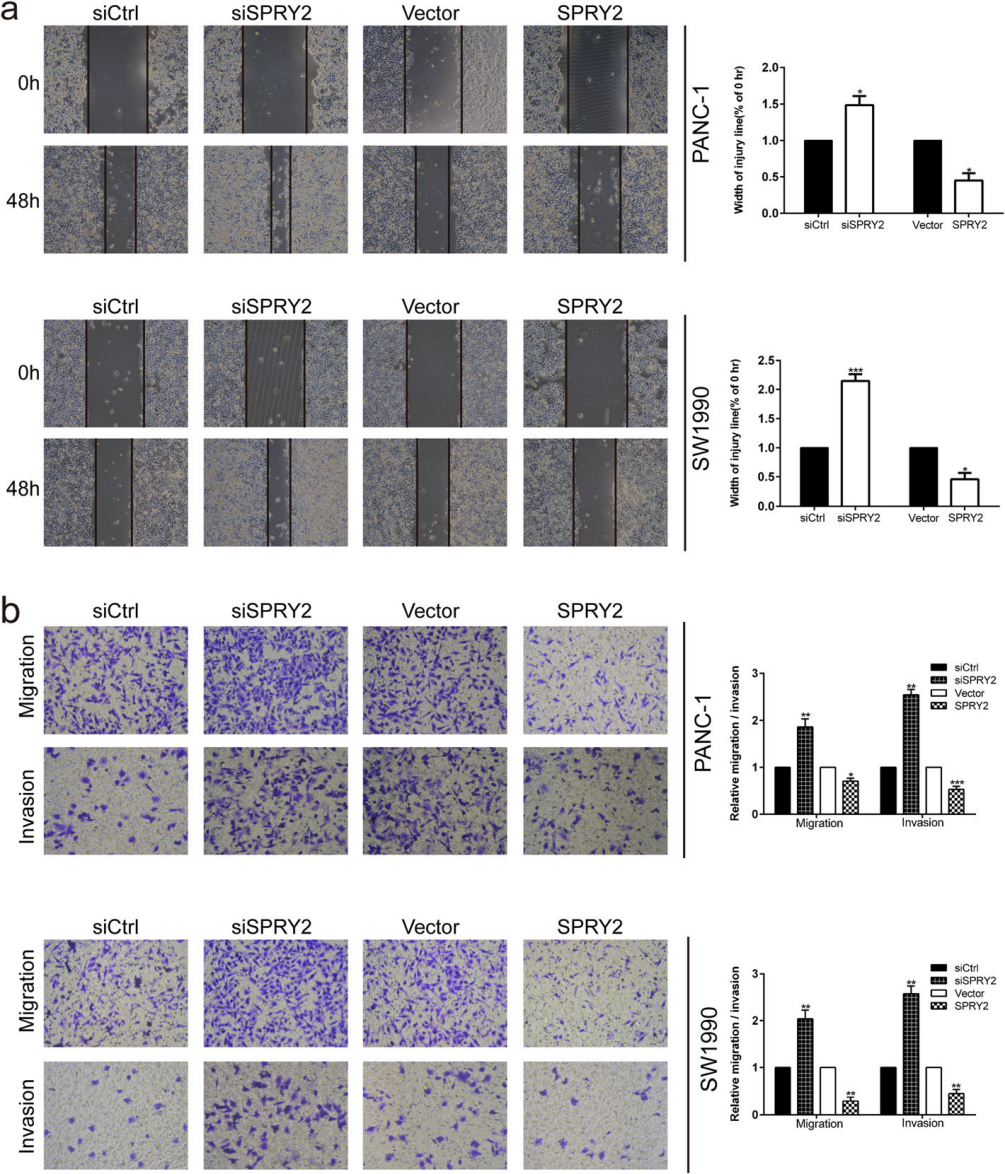
SPRY2 silencing promoted the migration and invasion of PDAC cells (**a**) Wound healing assay was carried out to investigate the migratory ability of PANC-1 and SW1990 cells. **b** Transwell assay was applied to assess the migratory and invasive capacities of PANC-1 and SW1990 cells. *P < 0.05, **P < 0.01, ***P < 0.001

**Fig. 6:**
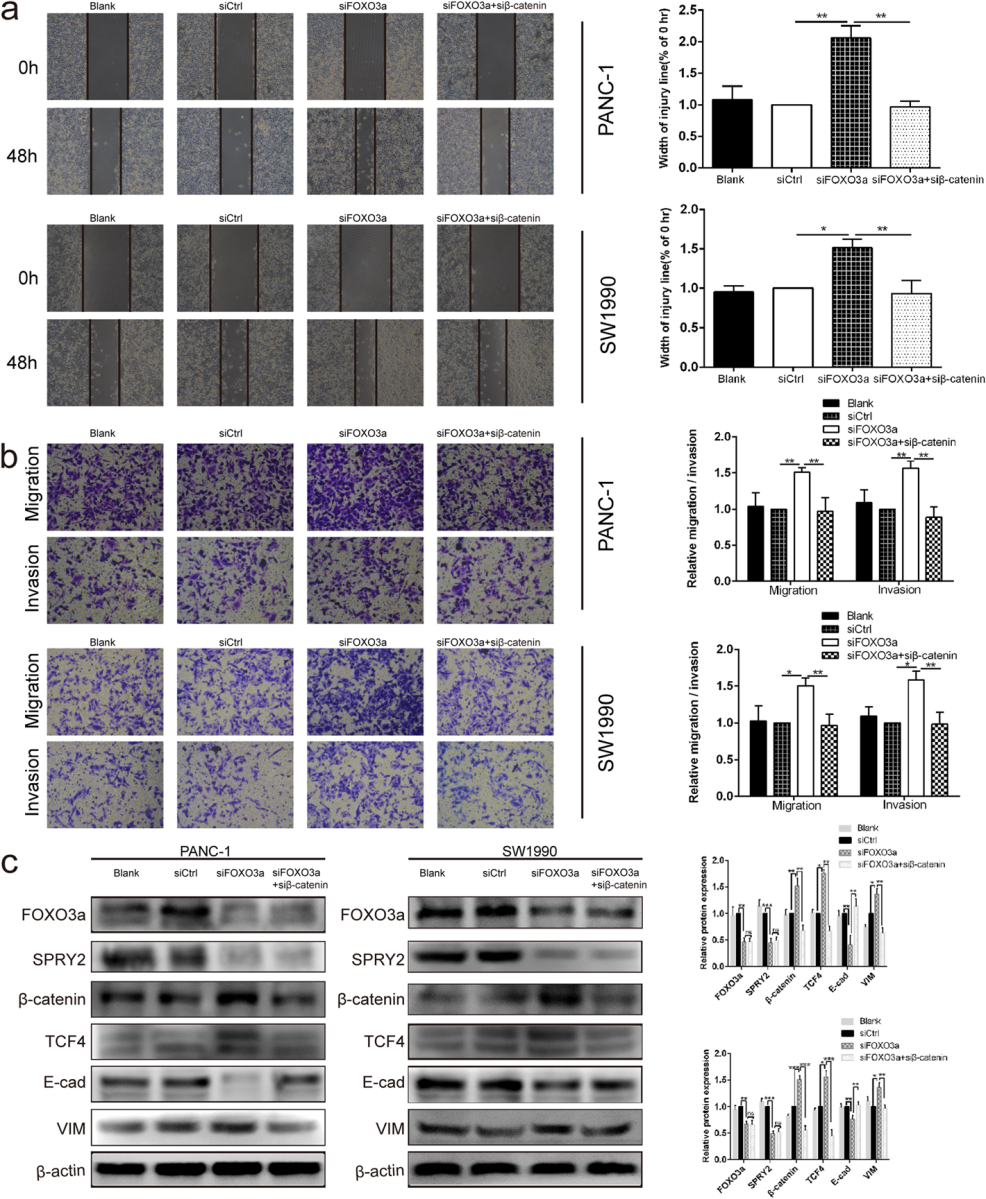
Silencing of β-catenin reversed the promotion effects of FOXO3a knockdown on EMT- associated migration and invasion of PDAC cells. **a** Wound healing assay was carried out to investigate the migratory ability of PANC-1 and SW1990 cells. **b** Transwell assay was applied to assess the migratory and invasive capacities of PANC-1 and SW1990 cells. **c** The protein expression of FOXO3a, SPRY2, β-catenin, TCF4, E-cad and VIM were detected in PANC-1 and SW1990 cells by Western blot. *P < 0.05, **P < 0.01, ***P < 0.001, ns non-significant

**Fig. 8:**
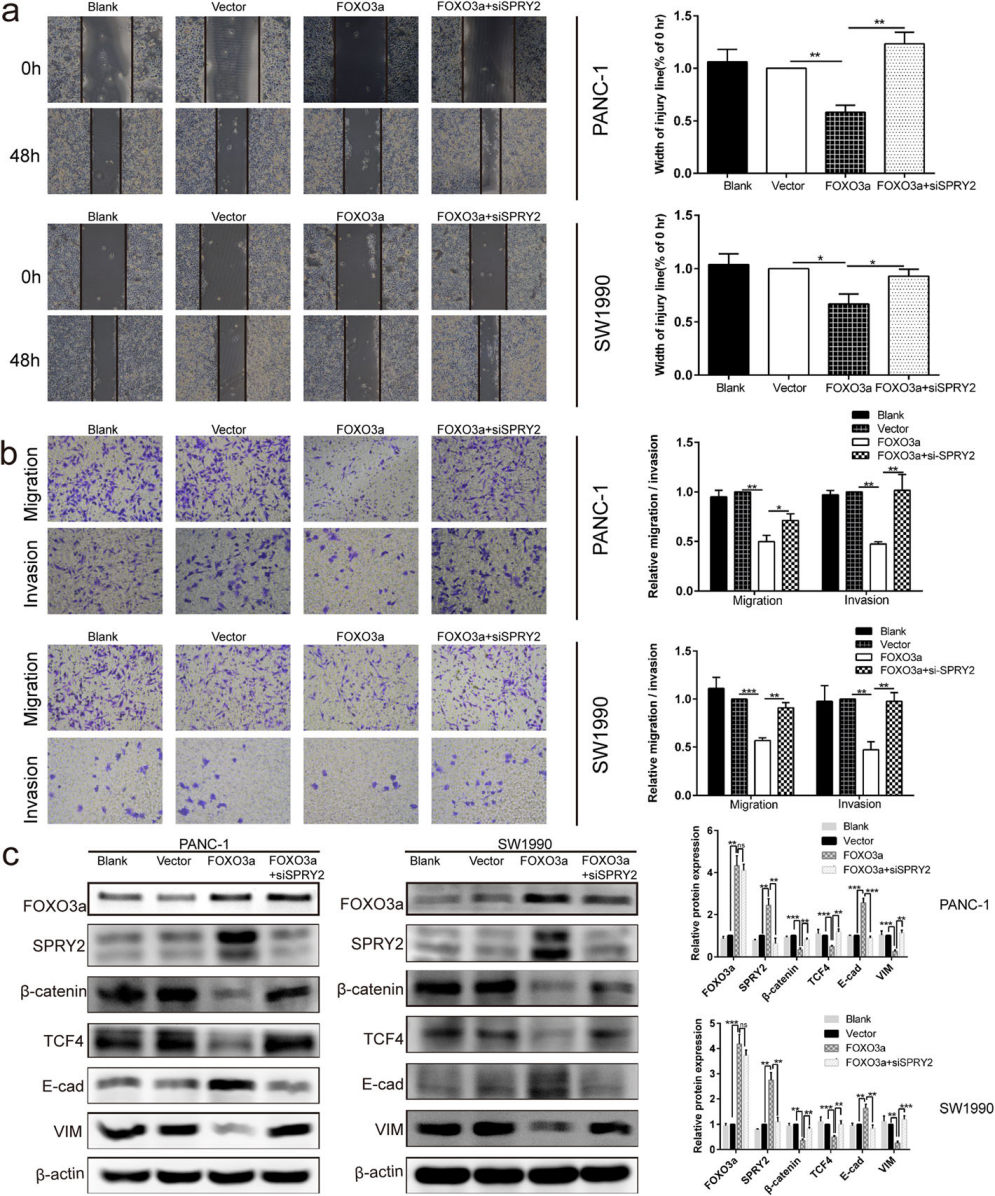
SPRY2 silencing reversed the suppressor effects induced by FOXO3a overexpression on EMT-associated migration and invasion of PDAC cells. **a** Wound healing assay was carried out to investigate the migratory ability of PANC-1 and SW1990 cells. **b** Transwell assay was applied to assess the migratory and invasive capacities of PANC-1 and SW1990 cells. **c** The protein expression of FOXO3a, SPRY2, β-catenin, TCF4, E-cad and VIM were detected in PANC-1 and SW1990 cells by Western blot. *P < 0.05, **P < 0.01, ***P < 0.001, ns non-significant

The authors provided the journal with the original data files. The corrected figures are given below. The correction does not have any effect on the results or conclusions of the paper. The original article has been corrected.
